# Laparoscopic Testicular Preservation in Adults with Intra-Abdominal Cryptorchidism: Is It Beneficial?

**DOI:** 10.1155/2012/329237

**Published:** 2012-11-22

**Authors:** Fábio César Miranda Torricelli, Marco Antonio Arap, Ricardo Jordão Duarte, Anuar Ibrahim Mitre, Miguel Srougi

**Affiliations:** Division of Urology, Hospital das Clinicas, University of São Paulo Medical School, Avenue Vereador Jose Diniz 3300, Conjunto 208, 04604-006 Sao Paulo, SP, Brazil

## Abstract

*Purpose*. To present the results in a midterm followup of laparoscopic testicular preservation in adults with intra-abdominal cryptorchidism. *Methods*. We analyzed 14 adult patients with cryptorchidism (19 testes) submitted to transabdominal laparoscopic evaluation and treatment of the condition. Data was collected retrospectively from a prospective database from August 2005 to May 2010. It analyzed patient age, affected side, procedure, mean operative time, mean hospital stay, postoperative testis position, intra- and postoperative complications, presence of malignancy in the removed testis, and midterm outcomes: size of the testis, development of tumors, and fertility. *Results*. Mean patient age was 29 (18–54) years. Thirteen (68.4%) testes were preserved. There were no intra- or postoperative complications. After a mean followup of 48.7 (20–64) months, all patients submitted to orchiopexy had the organs correctly positioned, although 2 testes were significantly smaller than before the procedure. No patient presented any signs of malignancy during the followup. Fertility was not preserved in bilateral cases. *Conclusion*. Laparoscopic testicular evaluation and eventual preservation are feasible and safe. In a midterm followup, testicular tumor is not a concern, and unfortunately, fertility may not be preserved in bilateral cases.

## 1. Introduction

Cryptorchidism is a condition that affects about 1% to 2% of boys, making it one of the most common congenital abnormalities of the genitourinary tract in infants. It is unilateral in 80% of cases [1, 2], and in approximately 20% of cases the testis is not palpable. When it is impalpable, half are intra-abdominal and half are absent or atrophic [2–5]. This disease is commonly diagnosed during childhood and prompt investigation and treatment are the rule. However, in some cases, usually in developing countries, diagnosis may be late due to ignorance or inaccessibility to health care and an adulthood presentation is seen.

Several diagnostic modalities have been used for localization and evaluation of impalpable testes. Ultrasound, computed tomography (CT), arteriography, and magnetic resonance have been used with variable success rates [6–8]. However, laparoscopy has become the preferential tool of investigation, as it allows diagnosis, evaluation, and treatment (orchiectomy or orchiopexy). In adults, in whom the risk of malignancy is a concern, an optimal evaluation is essential and laparoscopy provides it. Although, the most commonly procedure done is the orchiectomy, testicular preservation may be possible. Herein, we studied an adult population submitted to laparoscopic evaluation for intra-abdominal cryptorchidism. Testicular identification and the feasibility of laparoscopic preservation were the aims of our analysis. In addition, we sought to analyze if testes preservation was beneficial to the patient, in terms of testis size, tumor development, and fertility. We present the results of a midterm followup of laparoscopic testicular preservation in adults with intra-abdominal cryptorchidism.

## 2. Material and Methods

 Data was collected retrospectively from a prospectively maintained database from August 2005 to May 2010. In our institution, we have an electronic chart of all patients submitted to any kind of intervention in the urology division with demographic and disease-related data. This chart is prospectively maintained, and it was used for acquisition of our data. Laparoscopic orchiopexy and laparoscopic orchiectomy were the keywords searched. Fourteen adult patients with impalpable cryptorchidism submitted to transabdominal laparoscopic evaluation and treatment of the condition were analyzed. A total of 19 impalpable testes were found. All patients were previously submitted to ultrasound to evaluate the presence of testis in inguinal canal. One patient had a CT identifying an intra-abdominal left testis, and one patient had a magnetic resonance identifying an intra-abdominal left testis. Both evaluations were performed elsewhere before these patients were referred to our clinic. 

 Laparoscopy was performed with a 30° laparoscopy scope with the patient in Trendelenburg position. All patients had a foley and orogastric tube positioned after general anesthesia. A three-port technique was used for patients with unilateral cryptorchidism: a 10 mm umbilical port for the camera, a 10 mm port in the right or left iliac fossa, and a 5 mm port in the suprapubic region. Four ports were used for cases of bilateral cryptorchidism. Testis position was initially identified, and decision on the next step was made intraoperatively. Intraoperative biopsy was not done in any case, and this was always according to surgeon discretion. All removed testicles were sent to postoperative histopathological examination. A first-stage Fowler-Stephens (testicular vessel double clipping without section) was done for patients with high intra-abdominal testis (>2.0 cm above internal inguinal ring), while single-stage orchiopexy was performed for testes localized <2.0 cm of the internal inguinal ring. A wide and careful dissection of testicular vessels was always performed up to the renal hilum. A trocar through the scrotum or a small inguinotomy was used to help in testicular positioning into the scrotum. If the passage through inguinal canal was not possible, a subcutaneous tunnel was done. There was no rule to relieve vessels tension, except for a large dissection. Atrophic (<2.0 cm diameter) testes were resected as well as testes that no reach a reasonable position. All resected testes were submitted to histopathological examination. Six months after the first-stage Fowler-Stephens, the second-stage was performed.

 Sperm count was not routinely done for cases of unilateral cryptorchidism because patients did not present any concern with fertility when they came to our institution. In cases of bilateral cryptorchidism, we always tried to preserve at least one testis, and sperm count was routinely done.

 Data analyzed were patient age, affected side, procedure/technique performed, mean operative time, mean hospital stay, post-operative testis position, intra- and postoperative complications, presence of malignancy in the removed testis, and midterm outcomes: size of the testis, development of tumors, and fertility.

## 3. Results

 Mean patient age was 29 (18–54) years. Median age was 25.5 years. Five (35.7%) patients had left-side cryptorchidism, four (28.6%) right-side, and five (35.7%) bilateral. A total of 19 testes were evaluated. Testis identification was immediate in all cases, and all organs were intra-abdominal. Thirteen (68.4%) testes were preserved. Six testes were resected because of small size (<2.0 cm diameter) or difficult dissection. Two of these patients had bilateral cryptorchidism, and both testes were removed (one case had a seminoma in situ in the biopsy and the other case had both testes too small). Both patients were young men (23 and 26 years old), they did not have kids, and until now did not present any concern about that. The others three patients with bilateral cryptorchidism were treated as follows: one case with first-stage Fowler-Stephens in one side and single-stage orchiopexy in the other one; two cases with bilateral single-stage orchiopexy.

 Overall, three patients were treated with first-stage Fowler-Stephens, and all the others (10 testes) were positioned in a single-stage orchiopexy. The second-stage Fowler-Stephens was performed in those three patients six months after the first procedure, and it was uneventful. No intra- or post-operative complications occurred. Mean operative time was 129 (60–210) minutes. Mean (range) hospital stay was 2.1 (1–3) days, as the patient was usually admitted one day before the surgery and discharged in the first post-operative day. The pathological evaluation of the six removed testes revealed no signs of malignancy, and a significant atrophy of testicular parenchyma, except for one case in which in situ seminoma was found (patient with bilateral disease).

 After a mean followup of 48.7 (20–64) months, all patients submitted to orchiopexy had the organs correctly positioned, although 2 testes were significantly smaller than before the procedure (<2.0 cm diameter). These patients with atrophic testes are in close followup, and none of them presented any concern with testis size or fertility. In a midterm followup, no patient presented any signs of malignancy in physical examination or after ultrasound evaluation. Tumor makers were also done and always resulted normal. Regarding fertility, all five patients with bilateral disease presented with azoospermia. Patients with unilateral cryptorchidism were not routinely submitted to sperm count analysis. However, two patients demonstrated their desire to investigate fertility and had a sperm count within normal range.  Table 1 summarizes demographic data and intra- and postoperative outcomes. 

## 4. Discussion

 Untreated cryptorchidism in adults is a very rare condition, and the gold standard evaluation and management is still controversial. In this paper, we studied testicular preservation feasibility through laparoscopy and analyzed its consequences, such as testicular size, testicular tumor development, and fertility.

 It is known that early diagnosis and management of the undescended testis are needed to prevent testicular malignancy and preserve fertility [9, 10]. In men with bilateral cryptorchidism, one testis should be preserved, while in men with unilateral disease, orchiectomy can be done. However, we believe that this should not be a rule and, if testicular preservation is feasible, it should be offered to patients with relatively normal organs.

 Herein, we showed that testicular preservation in adults with intra-abdominal cryptorchidism is feasible and safe. We were able to preserve 68.4% of testes through laparoscopy depending on size, position, and contralateral testicular status. There were no intra- or immediate postoperative complications. Only two testes got smaller in a midterm followup. We interpreted this as atrophy secondary to the procedure, which is an expected result for the treatment of intra-abdominal cryptorchidism in adults. Corvin et al. [11] described their experience with laparoscopic management of adult cryptorchidism in 8 cases. In just one patient, a morphologically intact abdominal testicle was found and a first-stage Fowler-Stephens orchiopexy was performed. In all others cases, atrophic or vanishing testicles were found and resected. Vijjan et al. [12] reported better results in their experience with 14 adults with a mean age of 21 years. A total of 19 undescended testes were evaluated and 94.7% of the testes were intra-abdominal. Seven patients with unilateral undescended testes underwent laparoscopic orchiectomy, and laparoscopic-assisted orchiopexy was carried out in the remaining two patients. Five patients with bilateral undescended testes underwent laparoscopic orchiectomy on one side and laparoscopic-assisted orchiopexy on the other. Testicular preservation rate was 36.8%. The authors also concluded that laparoscopy is a safe and effective modality in the localization and management of adult undescended testes. In comparison to this series, we had a higher testicular preservation rate (68.4% versus 36.8%). It could be a selection bias because the keywords used in our research (laparoscopic orchiopexy and laparoscopic orchiectomy) did not include patients in whom no testes were found. However, to our knowledge, we present the largest series of preserved testes in adults with cryptorchidism. In addition, we are the first to report a followup of these patients over 48 months.

 In our study no patients presented any sign of testicular tumor in a midterm followup (48.7 months). All patients were routinely submitted to physical examination, ultrasound, and tumor markers evaluation as screening for testicular cancer. Regarding fertility, our analysis was impaired because patients with unilateral cryptorchidism were not routinely evaluated with sperm count, as usually this is not a concern in such patients. However, in patients with bilateral disease (five patients), even when one testis was preserved (three patients), azoospermia was the rule before the procedure. Therefore, we may conclude that testicular preservation in adults with bilateral cryptorchism should not be intended to reverse infertility. 

 Our paper has certain limitations. Although retrospective in nature, cryptorchidism in adults is a rare condition and large prospective series are difficult to be performed. We also did not evaluate the pre- and postoperative hormone levels of our patients however, it was not the purpose of our study, and future research will address that issue in a more comprehensive manner. 

## 5. Conclusion

 Laparoscopic testicular evaluation and eventual preservation are feasible and safe. In a midterm followup testicular tumor is not a concern, and, unfortunately, fertility may not be preserved in bilateral cases.

## Figures and Tables

**Table 1:** Demographic data and intra- and postoperative outcomes.

Patients	14

Mean age	29 (18–54) years

	19
Testes	5 bilateral
	5 right
	4 left

	13 (68.4%)
Preserved	3 first-stage Fowler-Stephens
	10 single-stage orchiopexy

	6 (31.6%)
Resected	4 due to small size
	2 due to difficult dissection

Mean operative time	129 (60–210) minutes

Hospital stay	2.1 (1–3) days

	11 well-positioned testes
Followup	2 smaller testes
	No signs malignancy
	Bilateral cryptorchidism = infertility
